# Elderberry-Based Multifunctional Prebiotic Systems Prepared via Spray Drying

**DOI:** 10.3390/biom15091289

**Published:** 2025-09-07

**Authors:** Anna Gościniak, Lidia Tajber, Piotr Szulc, Andrzej Miklaszewski, Tomasz M. Karpiński, Judyta Cielecka-Piontek

**Affiliations:** 1 Department of Pharmacognosy and Biomaterials, Poznan University of Medical Sciences, Rokietnicka 3, 60-806 Poznan, Poland; agosciniak@ump.edu.pl; 2 School of Pharmacy and Pharmaceutical Sciences, Trinity College Dublin, College Green, D02 PN40 Dublin, Ireland; ltajber@tcd.ie; 3 Department of Agronomy, Faculty of Agriculture, Horticulture and Biotechnology, Poznań University of Life Sciences, Dojazd 11, 60-632 Poznan, Poland; piotr.szulc@up.poznan.pl; 4 Faculty of Materials Engineering and Technical Physics, Institute of Materials Science and Engineering, Poznan University of Technology, M. Skłodowska-Curie Square, 60-965 Poznan, Poland; andrzej.miklaszewski@put.poznan.pl; 5 Department of Medical Microbiology, Medical Faculty, Poznan University of Medical Sciences, Rokietnicka 10, 60-806 Poznan, Poland

**Keywords:** elderberry extract, spray drying, galactooligosaccharides (GOS), chitooligosaccharides (COS), polyphenols, anthocyanins, degradation kinetics, silica

## Abstract

Elderberry (*Sambucus nigra* L.) is recognized as a rich source of anthocyanins and other bioactives with antioxidant and antidiabetic potential, and is increasingly explored as a functional ingredient in nutraceuticals. However, cultivar-dependent variability can strongly influence chemical composition and bioactivity, underscoring the need for careful selection of plant material prior to formulation. In the present study, twelve genotypes of elderberry were compared in terms of total polyphenols, antioxidant activity, and antiglycation potential. Based on the overall profile, ‘Samyl 1’ was advanced to formulation trials. Spray-dried carrier systems were produced using galactooligosaccharides (GOS) or chitooligosaccharides (COS), with or without colloidal silica. GOS-based powders retained anthocyanins at levels approaching theoretical values and exhibited superior thermal stability, as evidenced by differential scanning calorimetry, thermogravimetric analysis, and degradation-kinetic modeling, whereas COS matrices provided less effective stabilization. Incorporation of silica significantly enhanced technological properties, improving recovery, reducing agglomeration, and increasing flowability, without compromising anthocyanin content. All powders displayed low moisture (2.5–7.1%), favorable morphology, and preserved functional activity, aligning with stability requirements for shelf-stable plant extracts. Overall, the study demonstrates that strategic cultivar selection combined with GOS–silica carrier systems enables the production of stable elderberry powders that maintain high anthocyanin content and bioactivity. Such multifunctional ingredients couple prebiotic functionality with efficient delivery of polyphenols, highlighting their potential in nutraceutical and pharmaceutical formulations.

## 1. Introduction

Elderberry (*Sambucus nigra* L.) is a widely recognized medicinal plant known for its diverse health-promoting properties [1,2]. While the flowers of *S. nigra* have been extensively studied for their medicinal properties, the fruits also possess remarkable health-promoting potential due to their high content of anthocyanins and polyphenolic compounds [3]. Its fruits are particularly rich in bioactive compounds, especially anthocyanins, with cyanidin-3-glucoside and cyanidin-3-sambubioside being the predominant constituents [4]. Elderberry-derived polyphenols exhibit a broad spectrum of biological activities, including strong antioxidant potential and anti-glycation effects, which are of particular interest in the context of prevention of diabetes mellitus type-2. Elderberry phenolics, particularly anthocyanins and procyanidins, exhibit potent antioxidant and antidiabetic activities by effectively scavenging free radicals, inhibiting 15-lipoxygenase and xanthine oxidase, and showing superior α-amylase and α-glucosidase inhibition compared to acarbose, thereby enhancing the plant’s value as a functional food for diabetes prevention [5].

It is also known that the gut microbiota plays a critical role in the pathogenesis of type 2 diabetes by influencing glucose metabolism, intestinal permeability, and systemic inflammation [6]. Dysbiosis, characterized by a reduction in beneficial microbial populations, has been associated with impaired insulin sensitivity and an increased risk of metabolic disorders [7]. Individuals with type 2 diabetes often exhibit gut dysbiosis, including reduced microbial diversity and specific shifts in bacterial populations, such as decreased levels of *Firmicutes* and *Clostridium butyricum*, and an altered *Bacteroidetes* to *Firmicutes* ratio, which is associated with hyperglycemia [6,8,9]. This microbial imbalance promotes systemic inflammation, partly through increased levels of circulating lipopolysaccharides (LPS), which activate the TLR4 receptor and downstream inflammatory pathways, ultimately impairing insulin signaling [10,11,12]. On the other hand, microbiota-derived short-chain fatty acids (SCFAs), like butyrate, support intestinal barrier integrity and stimulate the release of GLP-1, enhancing insulin sensitivity and modulating inflammation. Therefore, gut microbiota composition and function are closely linked to the onset and progression of T2DM, making it a promising target for future dietary and therapeutic strategies.

Prebiotics are non-digestible carbohydrates that promote the growth of beneficial bacteria in the colon, mainly *Bifidobacterium* and *Lactobacillus* [13]. Galactooligosaccharides (GOS) are well-known prebiotics composed of galactose units with a glucose residue. They are not broken down in the upper gastrointestinal tract and are fermented by gut microbiota in the large intestine. Chito-oligosaccharides (COS), obtained from chitosan, also act as prebiotics and may support intestinal health through additional antimicrobial and anti-inflammatory properties. COS exhibit multiple bioactive properties and hold significant promise for applications in biomedicine and functional foods. Recent studies have demonstrated that COS can beneficially modulate gut microbiota composition, enhance SCFA production, and exert anti-inflammatory effects. In a neonatal necrotizing enterocolitis (NEC) rat model, COS supplementation improved survival rates, reduced intestinal damage, and downregulated pro-inflammatory cytokines. Moreover, COS increased the abundance of beneficial bacterial genera such as *Akkermansia*, *Bacteroides*, and *Clostridium*, both in vivo and in vitro, where COS fermentation also led to elevated levels of 3-hydroxybutyrate and γ-aminobutyric acid.

Recent investigations underline that the development of spray-dried systems increasingly relies on alternative carriers. Ben-Othman et al. [14] demonstrated that proteins derived from hemp, canola, and flax seeds can successfully replace conventional whey protein, yielding powders with comparable morphology and stability. Kucharska-Guzik et al. [15] showed that inulin represents a viable substitute for maltodextrin in the encapsulation of *Cistus creticus* polyphenols, while maintaining both antioxidant activity and technological performance. In line with this trend, Pashazadeh et al. [16] reported that the choice of drying technique and carrier material strongly affects polyphenol retention and bioaccessibility in okra flower extracts, with maltodextrin proving particularly effective in stabilizing quercetin and epicatechin. Furthermore, Žugić et al. [17] highlighted the dual role of pectin, which not only enhanced the encapsulation efficiency of green tea catechins and caffeine but also contributed intrinsic antioxidant and hypoglycemic properties, thereby extending the functional potential of the obtained powders. Collectively, these studies illustrate a shift towards the use of plant-based and multifunctional carriers, emphasizing both sustainability and the preservation of bioactive compounds in spray-dried formulations. Despite proven biological activity, the formulation of oligosaccharides into stable, functional delivery systems remains a technological challenge. Spray drying has emerged as a promising method to encapsulate and stabilize these compounds, particularly when combined with bioactive plant extracts such as elderberry. Recent studies have begun to explore their potential in this area, demonstrating promising results in both the stabilization of bioactive compounds and the development of functional powders. Similarly, Sosa et al. [18] demonstrated that galactooligosaccharides (GOS) combined with maltodextrin function effectively as thermoprotectants for *Lactiplantibacillus plantarum*, with the protective capacity closely linked to the matrix’s thermophysical properties and glass transition behavior (Tg).

Furthermore, Miravet et al. [19] reported the use of resistant dextrin and fructooligosaccharides (FOS) as effective drying aids in the spray drying of pomegranate juice, demonstrating their ability to reduce stickiness and preserve bioactivity. Resistant dextrin, in particular, was identified as the most efficient carrier, requiring minimal amounts to achieve free-flowing, rehydratable powders with preserved functional properties during storage. These examples highlight the growing interest in incorporating oligosaccharides as functional components in spray-dried systems, paving the way for innovations in the production of health-oriented food ingredients.

While encapsulation of plant polyphenols with carbohydrate-based carriers has been extensively studied, there is, to the best of our knowledge, a gap in the literature regarding direct, head-to-head comparisons of different prebiotic oligosaccharides within the same experimental framework [20]. In particular, no previous work has systematically evaluated and contrasted GOS and COS with respect to their efficiency in protecting anthocyanins, maintaining antioxidant capacity, and determining key powder properties such as morphology, particle size, and thermal stability. Given the limited application of oligosaccharides as spray-drying carriers and the growing interest in multifunctional powders with both antioxidant and prebiotic activity, this study aimed to develop and evaluate novel spray-dried systems based systems on elderberry extract with GOS or COS.

## 2. Materials and Methods

### 2.1. Materials

The plant material used in the study consisted of dried fruits of various black elderberry cultivars: (1) Samyl, (2) Samyl 1, (3) Obelisk, (4) Sambo, (5) Golden Hydrid, (6) Bez koralowy, (7) Haschberg, (8) Sampo, (9) Black Tower, (10) Black Beauty, (11) Haschberg 1, and (12) Bez dwubarwny. The black elderberry fruits were air-dried in a dry, well-ventilated area. The plant material for the analysis was sourced from the experimental station of the Research Centre for Cultivar Testing (COBORU) in Słupia Wielka (Poland).

Galactooligosaccharides (GOS) were supplied by Chemat (Gdańsk, Poland), chitosan oligosaccharides (COS) were obtained from PolAura (Warsaw, Poland). All solvents and reagents used were of analytical or HPLC grade. Aeroperl^®^ 300 Pharma (colloidal silica) was supplied by Evonik Industries (Essen, Germany). All chemical reagents, including Folin–Ciocalteu reagent, sodium carbonate, 2,2-diphenyl-1-picrylhydrazyl (DPPH), phosphate-buffered saline (PBS), and standards such as cyanidin 3-glucoside and cyanidin 3-sambubioside chloride, were purchased from Sigma-Aldrich (St. Louis, MO, USA). Solvents used for HPLC analysis (methanol, formic acid and acetonitrile) were of HPLC grade and purchased from POCH S.A. (Gliwice, Poland).

### 2.2. Optimization of Extraction

The extraction was designed to evaluate the effect of selected factors on the response variable. The studied parameters included methanol content in the extraction solvent (%, *v*/*v*), time (min), and the solvent-to-solid ratio (mL/g). In total, 15 extracts were obtained, each differing in the levels of the tested parameters. Other extraction conditions—namely the raw material weight (500 mg), extraction temperature (50 °C), and the number of extraction cycles (three repetitions), were kept constant for all samples. According to the experimental design (Table 1), 15 extracts were prepared. For each extract, 500 mg of the material was weighed into an Erlenmeyer flask, and a solvent (water, methanol, or a methanol–water mixture) in a volume of 10, 30, or 50 mL was added. The extraction solvent also contained 1% formic acid (HCOOH). The material was extracted three times in an ultrasonic bath at 50 °C for 15, 52.5, or 90 min, depending on the experimental run. After each extraction cycle, the plant material was filtered using a vacuum filter, and the residue was re-extracted with fresh solvent. After three extraction cycles, the combined extracts were brought to the appropriate final volume with solvent. The extracts were transferred to 50 mL falcon tubes, labeled according to Table 1, and stored at 2–8 °C until further analysis. The response variable in the experimental design was the total polyphenol content in the extracts, quantified by the Folin–Ciocalteu spectrophotometric method [21]. Based on optimization, optimal extraction conditions were selected, and extracts were made from elderberry varieties.

### 2.3. Antioxidant Activity

The antioxidant capacity of the tested extracts was assessed using two in vitro assays, each based on a different reaction mechanism. The Ferric Reducing Antioxidant Power (FRAP) method evaluates the reducing power of antioxidants by measuring their ability to reduce metal ions to lower oxidation states. The 2,2-Diphenyl-1-picrylhydrazyl (DPPH) assay is based on the scavenging of synthetic, stable free radicals by antioxidant compounds.

#### 2.3.1. Trolox Standard Curve

Calibration curves were established for Trolox, which served as the standard compound in all four antioxidant assays. A stock solution was prepared by dissolving 2 mg of Trolox in 1 mL of dimethyl sulfoxide (DMSO). The solution was then diluted 10-fold with water. Serial dilutions of the working solution were prepared to generate standard concentrations ranging from 100 to 800 µg/mL.

#### 2.3.2. Ferric Reducing Antioxidant Power (FRAP) Assay

The FRAP method measures the reduction of Fe(III) to Fe(II) in the presence of 2,4,6-tri(2-pyridyl)-s-triazine (TPTZ), forming a blue complex with maximum absorbance at 593 nm. The reagent was prepared from acetate buffer (300 mM, pH 3.6), TPTZ solution (10 mM in 40 mM HCl), and FeCl_3_ solution (20 mM) in a 10:1:1 ratio. Each well of a 96-well plate received 25 µL of extract and 175 µL of FRAP reagent. After 30 min of incubation at 37 °C, absorbance was measured at 593 nm (Multiskan GO 1510, Thermo Fisher Scientific, Vantaa, Finland). The antioxidant capacity of samples was expressed as milligrams of Trolox equivalents per gram of dry weight (mg Trolox/g DW).

#### 2.3.3. Diphenyl-1-Picrylhydrazyl (DPPH) Assay

A 0.2 mM solution of DPPH was prepared in methanol and protected from light. In each well of a 96-well plate, 25 µL of the test extract was added to 175 µL of the DPPH solution. The plate was incubated in the dark at room temperature for 25 min, and the absorbance was measured at 517 nm. The free radical scavenging capacity (%) of the test extracts and the standard sub-stance was calculated from the following Equation:

$${DPPH}_{scavenging\;activity}\left(\%\right)=\frac{A_{0}-A_{1}}{A_{0}}\times 100\%$$

where A_0_ is the absorbance of the control, and A_1_ is the absorbance of the sample. The antioxidant capacity of samples was expressed as milligrams of Trolox equivalents per gram of dry weight (mg Trolox/g DW).

### 2.4. Protein Glycation Assay

Briefly, 50 μL of protein solution (100 mg gelatine in 5 mL of distilled water) was added to 10 μL of glyceraldehyde solution (222 mg in 5 mL distilled water) in black microplates. The plate was then sealed and incubated at 37 °C for 24 h. After incubation, 40 μL of the plant extract (3.55 mg/mL) was added. A blank containing distilled water instead of the extract served as a negative control (untreated), while aminoguanidine (100 μM) was used as a positive control. The fluorescence was then measured at 370 nm (excitation) and 440 nm (emission). The activity was presented as the percentage of inhibition compared with the control sample, which was set as 0% inhibition.

### 2.5. HPLC Analysis

Qualitative and quantitative analyses of the prepared extracts were performed using a previously developed and validated high-performance liquid chromatography method with diode-array detection (HPLC-DAD). The analyses were conducted on a Shimadzu LC-2050C system (Shimadzu, Kyoto, Japan) equipped with a DAD detector. Chromatographic separation was achieved on a LiChrospher RP18-5 column (4.6 mm × 250 mm, 5 µm particle size) (Merck, Darmstadt, Germany). The mobile phase consisted of 5% formic acid in water (solvent A) and 5% formic acid in methanol (solvent B), delivered at a flow rate of 0.8 mL/min. The total run time was 30 min using a gradient elution as follows: 0–15 min, B = 15–55%; 15–20 min, B = 55–65%; 20–25 min, B = 65–70%; 25–27 min, B = 70–75%; 27–30 min, B = 75–15%. Detection was carried out at 520 nm, and the column temperature was maintained at 35 °C throughout the analysis.

### 2.6. Lyophilization

The selected extract, corresponding to the best-performing cultivar, was freeze dried using a Lyoquest −85 freeze dryer (Telstar, Eden Prairie, MN, USA). The process was conducted for 5 days at −85 °C and 0.2 mPa to ensure complete dehydration. The resulting dry mass was ground in an agate mortar to obtain a homogenous powder. The lyophilized samples were stored in tightly sealed Falcon tubes at 4–8 °C throughout the study.

### 2.7. Spray Drying

A 4 g mixture of plant extract (ELD) and oligosaccharide was dissolved in 100 mL of deionized water under continuous stirring to obtain a clear solution. The solution was then spray-dried using a Büchi B-290 Mini Spray Dryer (Büchi Labortechnik AG, Flawil, Switzerland) operating in open mode with compressed nitrogen as the drying medium. The spray drying was performed with an inlet temperature of 160 °C, which resulted in an outlet temperature between 70 and 80 °C. The aspirator was set to 90% to ensure efficient separation, and the feed solution was delivered at a pump rate of 20% (5 mL/min). The composition of the obtained systems is presented in Table 2.

### 2.8. Thermogravimetric Analysis

Thermogravimetric analysis (TGA) was conducted using a Q50 TGA V30 system (TA Instruments, New Castle, DE, USA). Samples weighing between 10.0 and 15.0 mg were placed in platinum pans and heated from 25 °C to 250 °C at a heating rate of 10 °C/min under a nitrogen atmosphere. The analysis was performed to evaluate the thermal stability and decomposition profile of the tested materials.

### 2.9. Morphological Characteristics

The surface morphology of the spray-dried powders was evaluated using a VHX-7000 digital microscope (Keyence, Osaka, Japan) equipped with high-resolution optical imaging and digital depth composition. Images were captured in 16-bit high dynamic range mode. Each final micrograph was generated by stacking 25 images along the Z-axis using automated focus analysis to ensure sharpness throughout the depth of field.

### 2.10. Particle Size

Particle size and distribution measurements were carried out using a laser diffraction analyzer (Mastersizer 3000, Malvern Instruments, Malvern, UK) with three replicate measurements (*n* = 3). The dry powder samples were dispersed using the Malvern Aero S module, fitted with a micro-volume tray, and operated at an air pressure of 3 bar. During measurement, an obscuration level between 0.5% and 6% was maintained, with a vibration feed rate set to 75%. Prior to data collection, a pressure titration test was performed to ensure the applied air pressure did not cause particle breakage or degradation. This involved analyzing particle sizes under varying air pressures to identify the threshold at which structural damage to the particles occurred. Data acquisition and particle size distribution analysis were conducted using the Mastersizer 3000 software (version 3.63).

### 2.11. Density

True density was measured using helium (99.995% purity) with a Micromeritics AccuPyc 1330 pycnometer (Norcross, GA, USA).

### 2.12. ATR-FTIR Spectroscopy

Infrared spectra in the mid-infrared (MIR) range (400–4000 cm^−1^) were recorded using an IRTracer-100 spectrophotometer equipped with a QATR accessory featuring a diamond ATR crystal (Shimadzu, Kyoto, Japan). Each spectrum was acquired with a resolution of 4 cm^−1^, averaging 100 scans per sample to ensure a high signal-to-noise ratio. Data collection and preliminary processing were carried out using LabSolution IR software (version 1.86 SP2, Shimadzu). The final spectral data and theoretical outputs were analyzed and visualized using Origin 2021b software (OriginLab Corporation, Northampton, MA, USA).

### 2.13. Differential Scanning Calorimetry (DSC)

DSC analysis was performed using a Mettler Toledo 821e (Greifensee, Switzerland) with a LabPlant RP-100 (Manchester, UK) cooling system and nitrogen as the purge gas. Hermetically sealed 40 μL aluminum pans with three vent holes were used, with sample weights of 3–6 mg. The system was calibrated with indium and zinc, and all measurements were conducted at a 10 °C/min heating rate. Data analysis was carried out using Mettler Toledo STARe software (version 6.10) on Windows NT.

### 2.14. Degradation Kinetics

The stability of elderberry extract in spray-dried formulations was evaluated under accelerated thermal conditions to assess the protective capacity of selected carrier systems. Samples containing GOS or COS were subjected to thermal degradation at 85 °C for 4 h. The quantification of anthocyanins was performed by high-performance liquid chromatography (HPLC), focusing on two major compounds, cyanidin-3-glucoside and cyanidin-3-sambubioside, which were summed to determine the total anthocyanin content. Degradation kinetics were modeled using a first-order reaction equation, and the corresponding degradation rate constants (k), correlation coefficients (r), and half-lives (t_0.5_) were calculated to enable quantitative comparison of stability between formulations.

### 2.15. Statistical Analysis

The optimization of extraction parameters was performed using Statistica 13.3 (StatSoft Inc., Tulsa, OK, USA), applying response surface methodology based on the Box–Behnken design. All other statistical analyses were conducted using PQStat Software version 1.8.4.142 (2022, PQStat Software, Poznań, Poland). The normality of residuals was assessed using the Shapiro–Wilk test, and the Brown–Forsythe test was applied to verify the homogeneity of variances across groups. Differences between mean values were evaluated using one-way ANOVA, followed by Tukey’s post hoc test for multiple comparisons. If the homogeneity of the data was violated, the Games–Howell test was applied. Statistical significance was set at *p* < 0.05. Different lowercase letters indicate statistically significant differences between means within the same column.

## 3. Results and Discussion

### 3.1. Optimization of the Extraction Procedure

**Figure 1:**
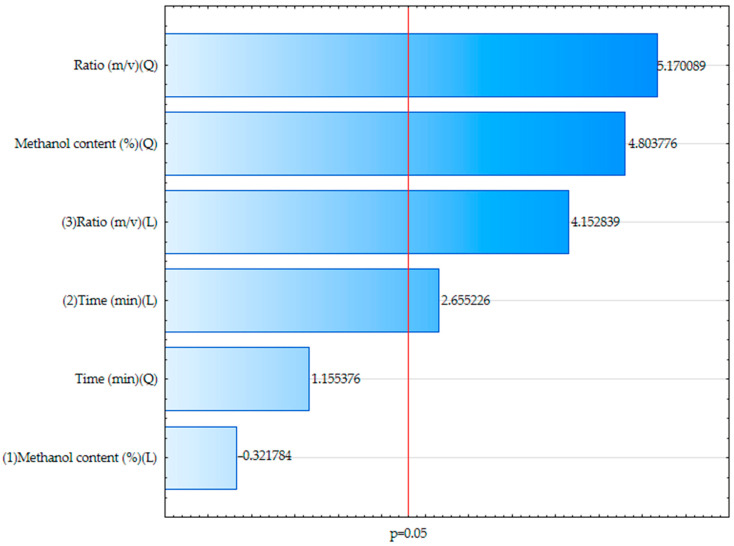
Pareto chart of standardized effects for variables affecting polyphenol content in the extract.

**Figure 2:**
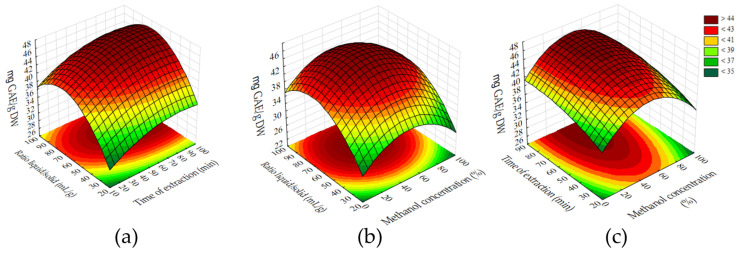
Response surface plots of total polyphenol content (TPC) as a function of extraction time and liquid/solid ratio (**a**), liquid/solid ratio and methanol concertation (**b**), liquid/solid ratio and methanol concertation (**c**).

The application of the Box–Behnken design proves advantageous for optimizing extraction processes, enabling effective maximization of both total polyphenol content and specific anthocyanins [22,23]. Based on the optimization conducted using the Box–Behnken design, the most significant factors influencing the extraction efficiency were identified through the Pareto chart (Figure 1). The extraction ratio (m/v) had the most pronounced effect, followed by the methanol concentration and extraction time (*p* < 0.05). This is confirmed by the standardized effects and their statistical significance. The response surface methodology (Figure 2) illustrates the interactions between these variables and their combined impact on the polyphenol yield. The regression model showed good predictive capability, with an R^2^ of 0.8986 and an adjusted R^2^ of 0.8225, indicating that approximately 90% of the variability in the total phenolic content could be explained by the model. The optimal extraction conditions were determined to be 48.86% methanol, 82 min of extraction time, and a solid-to-solvent ratio of 70.91 m/v. Under these parameters, the extract exhibited the highest concentration of polyphenolic compounds, as quantified by the Folin–Ciocalteu assay (TPC).

The optimized extraction conditions are consistent with values reported in other studies. Similar alcohol concentrations were found effective by Kim et al. [24] (40.9% ethanol), Domínguez et al. [25] (50%), Zao et al. [26] (63.8% methanol), and studies on grape byproducts (66% ethanol), confirming that alcohol content around 40 to 66 percent is commonly optimal for polyphenol extraction [27]. In contrast, temperature varies widely depending on the plant matrix and the goal of extraction. Reported optimal values range from 20 to 94 °C in previously mentioned studies. This variability reflects whether studies targeted anthocyanins specifically or aimed for a broader spectrum of compounds. In our study, a fixed temperature of 50 °C was initially selected. Extraction time also played an important role, with our optimal duration of 82 min falling within the broader range reported in the literature (15 to 93 min), depending on the compound type and plant matrix. In our case, the goal was to maximize total polyphenol content due to their diverse biological activity, which justified longer extraction time and moderate temperature. Fewer studies have investigated the effect of the solid-to-liquid ratio, yet in our research, this parameter has also been shown to be significant. Its strong influence on extraction efficiency highlights the importance of considering solvent volume when optimizing plant-based extractions.

### 3.2. Cultivars Comparison

Based on the optimization process, the extraction parameters were selected to maximize the polyphenol content in the final extract. The subsequent stage of the study involved comparing different elderberry cultivars to identify the one with the most favorable properties. The tested cultivars of black elderberry differed significantly in terms of antioxidant activity and the content of bioactive compounds.

#### 3.2.1. Active Compounds Content

Significant differences were observed among the elderberry cultivars in terms of anthocyanin composition and total polyphenol content (TPC) (Table 3). The predominant anthocyanins identified were cyanidin-3-glucoside and cyanidin-3-sambubioside. ‘Samyl 1’ and ‘Sampo’ showed the highest levels of cyanidin-3-sambubioside (0.846 ± 0.003 and 0.840 ± 0.004 mg/g DW, respectively), which were significantly higher than in other cultivars (*p* < 0.05). These two cultivars also exhibited elevated levels of cyanidin-3-glucoside and were among those with the highest TPC values.

The total polyphenol content was highest in ‘Samyl 1’ (57.75 ± 3.32 mg GAE/g DW), followed by ‘Sampo’ (49.64 ± 1.15 mg Gallic Acid Equivalent (GAE)/g DW) and ‘Sambo’ (48.13 ± 0.85 mg GAE/g DW), suggesting a strong correlation between anthocyanin content and overall polyphenolic richness. On the other hand, ‘Golden hybrid’ and ‘Obelisk’ showed the lowest levels of both anthocyanins and TPC, with cyanidin derivatives not detected (n.d.) in the ‘Golden hybrid’ cultivar. These results are consistent with previous studies, where cyanidin derivatives were reported as dominant anthocyanins in *Sambucus nigra* and closely linked to antioxidant activity. For example, Mikulic-Petkovsek et al. [28] demonstrated that elderberry fruits contain high levels of cyanidin-3-sambubioside, and their content varies strongly with genotype and ripening stage.

#### 3.2.2. Antioxidant Activity

**Figure 3:**
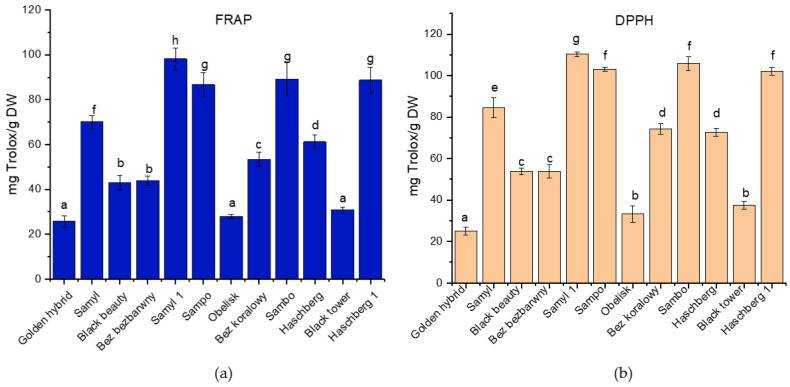
Antioxidant activity of extracts from different elderberry cultivars evaluated using (**a**) FRAP and (**b**) DPPH assays. Data are expressed as mean ± standard deviation (n = 5). Error bars indicate standard deviation. Different letters above the bars indicate statistically significant differences between means (*p* < 0.05; Tukey’s HSD test).

Elderberry fruits are recognized for their substantial antioxidant activity, primarily attributed to their high concentrations of phenolic compounds, such as anthocyanins and flavonoids. The presence of these compounds plays a vital role in combating oxidative stress, suggesting that they contribute to various health benefits associated with elderberry. The results demonstrated significant differences in antioxidant activity among the tested elderberry cultivars in both the FRAP and DPPH assays (Figure 3). The highest antioxidant capacity in the FRAP assay was observed for the ‘Samyl 1’ cultivar, followed by ‘Sampo’ and ‘Haschberg 1’, while the lowest activity was found in ‘Golden hybrid’, ‘Obelisk’, and ‘Black tower’.

Similarly, in the DPPH assay, ‘Samyl 1’ exhibited the strongest radical scavenging activity, followed by ‘Sampo’, ‘Sambo’, and ‘Haschberg 1’. The lowest DPPH values were recorded for ‘Golden hybrid’ and ‘Bez koralowy’. The observed differences were statistically significant (*p* < 0.05), indicating a substantial variation in antioxidant potential between cultivars, which may be attributed to differences in the content and composition of phenolic compounds. Previous studies have also confirmed the antioxidant properties of elderberry fruits, primarily attributed to their rich polyphenol content [29]. As shown by Csorba et al. [30], the antioxidant activity depends not only on the extraction method but also significantly on the cultivar and harvest year. Our findings support this, with ‘Samyl 1’ exhibiting the strongest activity in both FRAP and DPPH assays, correlating with its high anthocyanin and total polyphenol content. In contrast, cultivars like ‘Golden hybrid’ and ‘Obelisk’ showed the lowest antioxidant capacity, reflecting their lower contents of bioactive compounds. These results highlight the critical role of phenolic composition and genetic variability in determining antioxidant potential. Other studies also confirmed that elderberry has a notable concentration of anthocyanins, which are potent antioxidants. For instance, Ferreira et al. [31] confirmed that elderberries protect HepG2 and Caco-2 cells from oxidative stress. Additionally, measurements of antioxidant capacity using methods like ABTS, DPPH, and FRAP reveal that elderberry possesses notable activities—up to 137 µmol Trolox/g DW in ABTS, 109 µmol Trolox/g DW in DPPH, and 221 µmol Trolox/g DW in FRAP at full fruit maturity, demonstrating its strong radical-scavenging and -reducing potential [32].

#### 3.2.3. In Vitro Antidiabetic Activity

Advanced glycation end products (AGEs) are compounds formed through the reaction between sugars and proteins or lipids, which can lead to various negative health effects, including the progression of diabetes and cardiovascular diseases [33]. Recent studies have indicated that certain phenolic compounds may possess the capability to inhibit the formation of AGEs, presenting a potential avenue for the development of functional foods aimed at mitigating these health risks [34]. Elderberry is noted for its rich phenolic content, particularly flavonoids, which have garnered interest in the context of AGE inhibition.

**Figure 4:**
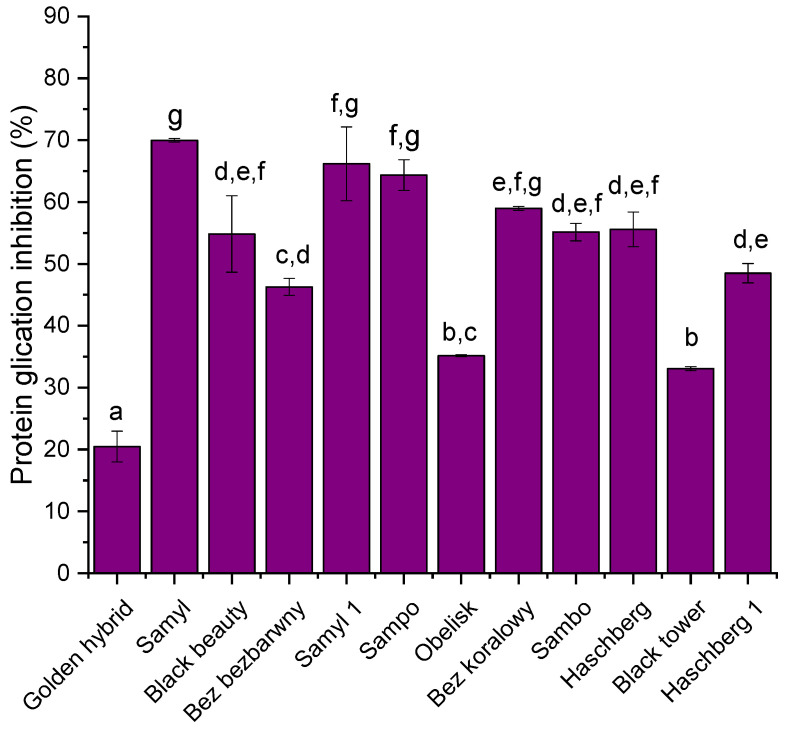
Inhibitory activity of elderberry fruit extracts from different cultivars against protein glycation. Data are expressed as mean ± standard deviation (n = 5). Error bars indicate standard deviation. Different letters above the bars indicate statistically significant differences between means (*p* < 0.05; Tukey’s HSD test).

All tested varieties showed potential for forming AGEs. The highest antiglycation activity was observed in the ‘Samyl’ cultivar, which inhibited protein glycation by nearly 70% (69.98 ± 0.29%), followed closely by ‘Samyl 1’ (66.18 ± 5.95%) and ‘Sampo’ (64.35 ± 2.48%) (Figure 4). These cultivars demonstrated significantly higher inhibitory potential compared to the rest of the samples (*p* < 0.05). Moderate activity was recorded for cultivars such as ‘Bez koralowy’ (58.98 ± 0.32%), ‘Haschberg’ (55.58 ± 2.81%), ‘Sambo’ (55.14 ± 1.40%), and ‘Black beauty’ (54.83 ± 6.17%). In contrast, the lowest antiglycation activity was found in the ‘Golden hybrid’ (20.47 ± 2.50%) and ‘Black tower’ (33.07 ± 0.31%) cultivars, indicating limited potential to inhibit the formation of advanced glycation end products (AGEs). The results confirm significant differences (*p* < 0.05) in antiglycation capacity between cultivars, which may reflect variations in the content and composition of phenolic and anthocyanin compounds known to interfere with glycation processes.

Although most antidiabetic studies on *Sambucus nigra* focus on flower extracts, exhibiting strong inhibition of carbohydrate-digesting enzymes (e.g., α-glucosidase, α-amylase) attributed to chlorogenic acid and quercetin derivatives, the fruit also deserves attention due to its rich polyphenol and anthocyanin profile [35]. In vitro and animal models have shown that elderberry fruit extracts exert antidiabetic effects, including improved glucose uptake, reduced insulin resistance, and antiglycation activity [36,37,38,39]. Polyphenols such as cyanidin-3-glucoside, quercetin, and chlorogenic acid, present in the fruits, were linked to strong antiglycation activity, preventing advanced glycation end-product (AGE) formation, a key mechanism in diabetes complications [40,41,42]. Although direct assays on fruit extracts are less common than those on flowers, our current data confirm significant antiglycation activity across cultivars, particularly ‘Samyl’ and ‘Sampo’. This aligns with prior evidence that elderberry phenolics can inhibit AGEs in food matrices. In summary, while *S. nigra* flowers remain the primary focus in antidiabetic research, the fruit’s bioactive compounds, notably polyphenols and anthocyanins, indicate its substantial potential in glucose regulation and antiglycation [43].

### 3.3. Spray-Drying Process Preparation of Elderberry Based Prebiotic Systems

#### 3.3.1. Recovery

Recovery yield is a key parameter reflecting the efficiency of the spray-drying process and the suitability of the carrier system. The recovery yields of spray-dried elderberry extract formulations are presented in Table 4. GOS-based systems showed higher recovery (up to 61.9%) compared to COS-based ones (as low as 46.3%). The addition of 15% of silica improved the yield in both systems.

Spray drying is a widely used encapsulation method that enables the production of stable, powdered forms of sensitive plant extracts. It offers several advantages, including scalability, short processing time, and improved product shelf life.

The recovery values obtained in this study are consistent with previous findings. Tonon et al. [44] reported recovery yields between 34.39% and 55.66% for açai juice spray-dried with maltodextrin. Similarly, Wilkowska et al. [45] found recovery rates ranging from 45.9% to 46.2% when spray drying blueberry juice using HP-β-CD and maltodextrin. Ravichandran et al. [46] achieved >60% recovery for anthocyanin-rich extracts using spray drying, confirming the expected efficiency range for fruit-derived systems.

These comparisons confirm that the recovery rates in our formulations are within the expected range for anthocyanin-rich plant materials and reflect efficient process performance.

**Table 4:** Recovery yield (%) of spray-dried elderberry extract powders formulated with GOS or COS carriers at different ratios, with and without 15% silica addition. Data are expressed as mean ± standard deviation (n = 2).

GOS Systems	Recovery (%)	COS Systems	Recovery (%)
ELD:GOS (1:1)	49.9 ± 2.3	ELD:COS (1:1)	47.5 ± 2.0
ELD:GOS (1:2)	61.9 ± 3.0	ELD:COS (1:2)	46.3 ± 3.2
ELD:GOS (1:3)	60.9 ± 3.8	ELD:COS (1:3)	48.9 ± 2.5
ELD:GOS (1:4)	59.9 ± 3.5	ELD:COS (1:4)	48.5 ± 3.6
ELD:GOS (1:1) + 15% Aeroperl	52.0 ± 1.5	ELD:COS (1:1) + 15% Aeroperl	52.2 ± 1.6
ELD:GOS (1:2) + 15% Aeroperl	58.6 ± 2.1	ELD:COS (1:2) + 15% Aeroperl	55.2 ± 2.0
ELD:GOS (1:3) + 15% Aeroperl	59.2 ± 2.1	ELD:COS (1:3) + 15% Aeroperl	58.5 ± 1.8
ELD:GOS (1:4) + 15% Aeroperl	58.7 ± 2.3	ELD:COS (1:4) + 15% Aeroperl	58.9 ± 2.2

#### 3.3.2. Moisture Content

Moisture content is a key indicator of powder quality, affecting shelf stability and handling properties. The moisture content was assessed by TGA in the range of 25–100 °C to quantify water loss. In our study, all formulations remained within the typical moisture range expected for spray-dried plant extracts, generally below 6% (Table 5). In the context of spray drying berry extracts, the moisture content of the final product is a significant parameter influencing the powder’s quality, shelf life, and application potential. Moisture levels below 10% are generally considered necessary to ensure microbiological stability and extend the shelf life of spray-dried plant powders.

Studies have demonstrated that moisture levels vary based on several factors, including the choice of carrier agents, drying temperature, and the characteristics of the berry extract itself. For instance, Romero-Román et al. [47] discuss the encapsulation of calafate berry extract using spray drying, noting that moisture content can significantly influence the retention of bioactive compounds during the drying process. The authors found that optimizing the spray-drying conditions resulted in a final moisture content range from approximately 3% to 5%, which is crucial for maintaining the stability of phenolic compounds over time. Their research highlights the importance of controlling moisture content to prevent degradation of these sensitive bioactive components. In a related study, González-Esparza et al. [48] investigated the moisture content of spray-dried powders derived from Murta berries, revealing that the lowest moisture content, 3.9%, was achieved when utilizing higher inlet temperatures (140 °C) combined with lower feed flow rates. This case exemplifies how varying these operational parameters affects the moisture retention and overall stability of the product. Further inquiries into different berry extracts reveal that the choice of carrier matrix can also influence moisture content. A study by Lourenço et al. [49] on pineapple peel extract indicated that the moisture content of spray-dried powders could range from 1.87% to 6.29% when using various wall materials like maltodextrin and inulin. The variations in moisture levels highlight how different encapsulation strategies can be tailored to achieve desired outcomes in powder characteristics.

#### 3.3.3. HPLC Analysis

The retention of anthocyanins following spray drying was evaluated by comparing the measured concentrations of cyanidin-3-sambubioside and cyanidin-3-glucoside with theoretical values calculated based on the dilution caused by the addition of prebiotic carriers (GOS or COS) and silica (Table 6). These theoretical values, presented in parentheses, represent the expected anthocyanin content, assuming no losses during processing. Across all formulations, the GOS-based systems exhibited minimal deviations from theoretical values, indicating high anthocyanin retention and stability during spray drying. The retention of cyanidin-3-sambubioside relative to the theoretical values derived from formulation composition did not fall below 93.9% for GOS-based systems and 71.3% for COS-based systems, whereas for cyanidin-3-glucoside it was not lower than 91.9% and 74.6%, respectively. The spray-drying inlet temperature of 160 °C used in this study is considered suitable for polyphenol-rich extracts and allows efficient water evaporation without inducing visible degradation of anthocyanins. However, the observed losses in COS-based systems suggest that temperature was not the only factor impacting the anthocyanin content. These findings highlight the importance of carrier composition and matrix structure in preserving thermolabile compounds during high-temperature drying. In most GOS formulations, the measured anthocyanin content closely matched the calculated values, particularly for cyanidin-3-sambubioside. This suggests that GOS provided a protective matrix that effectively preserved anthocyanins. In contrast, the COS-based systems showed more pronounced losses, with measured anthocyanin content falling below the expected theoretical values. These results indicate that COS may offer less protection for anthocyanins under the same drying conditions. In GOS-based systems, silica had no effect on anthocyanin retention during spray drying.

The process temperature used in the spray drying of berry extracts plays a crucial role in determining the quality and stability of the final product. Different studies have reported various inlet and outlet air temperatures that optimize the drying process while preserving the bioactive compounds present in the berries. Typically, spray drying of berry extracts involves inlet air temperatures ranging from 130 °C to 180 °C. For example, Ersus and Yurdagel [50] reported that for certain formulations, spray drying could effectively occur at air inlet temperatures up to 180 °C without adversely affecting the stability of the contained bioactive compounds. Numerous studies emphasize that the inlet and outlet temperatures during spray drying directly affect the retention of anthocyanins. For instance, Gaona et al. [51] observed high retention rates of monomeric anthocyanins in red wine extracts, with values consistently above 80% when employing inlet air temperatures between 140 °C and 160 °C. The choice of carrier material is also a critical factor that influences anthocyanin retention during spray drying. In studies involving maltodextrin and gum arabic, Silva et al. claimed that these materials created favorable chemical interactions, which helped retain anthocyanins during the drying process [52]. Moreover, Nguyen et al. [53] also noted that the type of encapsulating agent modified the efficacy of anthocyanin retention, highlighting the role of formulation in stabilizing these sensitive compounds during drying.

We attribute the superior performance of GOS to its stronger glass-forming tendency and lower water plasticization under our conditions, which together reduce molecular mobility and limit thermally driven degradation of anthocyanins [18]. In line with the framework proposed by Tymczyszyn et al. [54] who highlighted three complementary mechanisms for polyhydroxylated protectants (water replacement hydrogen bonding, vitrification-like immobilization that effectively raises Tg, and preferential exclusion/hydration), GOS appear to promote a lower-mobility amorphous matrix and more favorable sugar–pigment interactions than COS, thereby enhancing anthocyanins preservation during drying and storage. The higher anthocyanins retention observed for GOS-based systems compared to COS-based systems indicates that the selected carrier has a significant impact on preserving bioactive compounds during spray drying and subsequent storage. Given that anthocyanin content at the point of consumption determines the actual dose delivered to the consumer, these results suggest that GOS-based formulations may offer improved functional efficacy. Kumkum et al. [55] confirmed that the food matrix and processing conditions significantly influence anthocyanin stability and, consequently, their bioavailability in vivo, with improved stability often translating into greater absorption efficiency [1].

### 3.4. Physical and Morphological Properties of Spray-Dried Powders

#### 3.4.1. Morphological Comparison

**Figure 5:**
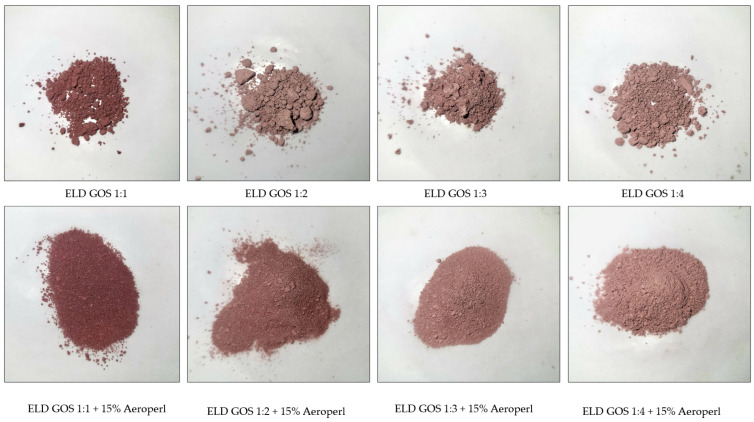
Visual appearance of elderberry extract–GOS systems with and without silica addition.

Spray-dried powders often tend to form agglomerates due to high surface energy, residual moisture, and cohesive interactions between particles. In the present study, morphological evaluation revealed clear differences in particle structure depending on the carrier type and the presence of silica. The visual comparison of the powders shows that the addition of 15% silica significantly improved the physical properties of the elderberry extract–GOS systems (Figure 5). Without silica (top row), the powders appear more heterogeneous, with visible agglomerates. In contrast, the samples containing 15% silica (bottom row) exhibit visibly enhanced powder structure being finer, more homogeneous, and better dispersed. This suggests that silica acts as an anti-caking and flow-enhancing agent by absorbing moisture and reducing interparticle interactions during spray drying.

**Figure 6:**
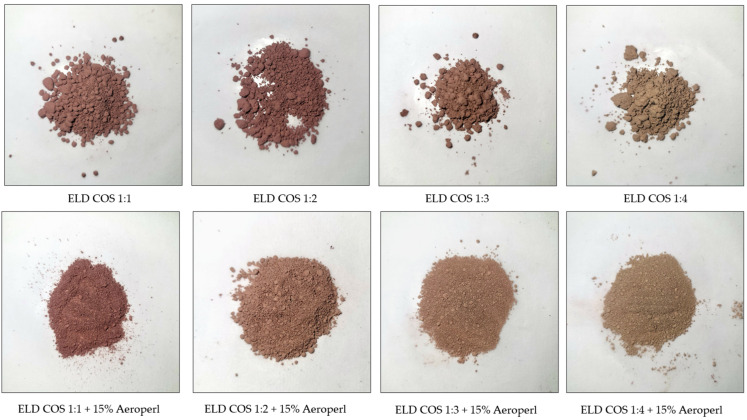
Visual appearance of elderberry extract–COS systems with and without silica addition.

Similar improvements in morphology were also observed for COS-based systems, where silica addition led to reduced agglomeration and more uniform particle distribution, despite their initially higher tendency to form agglomerates (Figure 6).

Studies indicate that the choice and proportion of carrier materials significantly influence the physical properties and morphology of spray-dried powders. The presence of stickiness, mainly due to sugars and polyphenols abundant in berry extracts, contributes substantially to agglomeration. Carriers such as maltodextrin or gum arabic are commonly used to enhance flowability and reduce agglomeration by forming a protective matrix that limits inter-particle adhesion [56]. However, in our preliminary trials, maltodextrin and gum arabic did not yield satisfactory morphology, likely due to insufficient encapsulation efficiency in this system. Conversely, silica is often incorporated not only to improve powder flow and dispersibility but also to enhance the solubility and stability of active compounds [57]. Our findings confirm that the absence of silica in GOS- and COS-based formulations leads to pronounced agglomeration, as observed in microscopic analysis. Thus, carrier selection, ratio, and inclusion of drying aids like silica are key factors shaping the final morphology of spray-dried plant-based powders, consistent with previous literature on the encapsulation of bioactive ingredients.

#### 3.4.2. Microscopic Analysis

**Figure 7:**
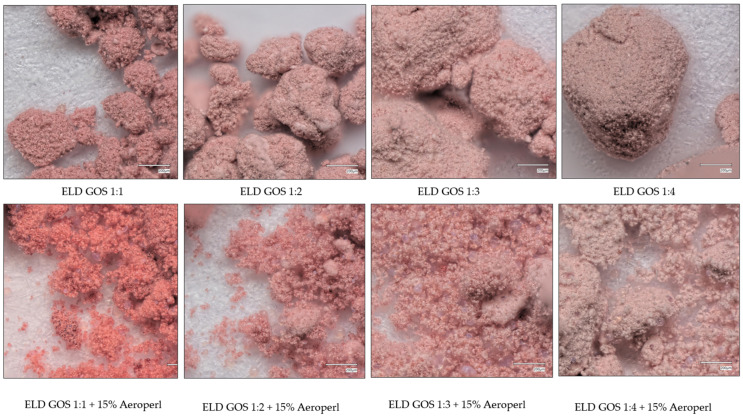
Microscopic morphology of ELD:GOS systems at different carrier ratios with and without silica addition.

**Figure 8:**
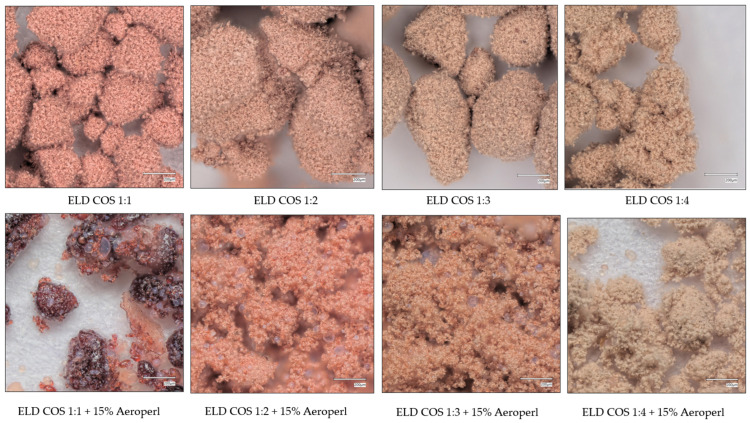
Microscopic morphology of ELD:COS systems at different carrier ratios.

The microscopic comparison of spray-dried powders shows that the addition of silica substantially improves particle morphology in both ELD:GOS and ELD:COS systems (Figure 7 and Figure 8). Without silica, powders show a high degree of agglomeration and poor dispersion, regardless of the carrier type or ratio. The incorporation of 15% silica leads to visibly more homogeneous and better separated particles, enhancing the structural integrity of the powders. This effect is consistent in both GOS- and COS-based formulations. However, in the ELD:COS 1:1 system, even with silica, noticeable agglomerates and visible fragments of the extract remain, indicating insufficient encapsulation and stabilization at this low carrier ratio. This highlights the limited protective capacity of COS at lower proportions, even in the presence of silica.

#### 3.4.3. Particle Size

Particle size is a critical parameter influencing the flowability, reconstitution, and packing behavior of spray-dried powders. The particle size distribution of the ELD:OS systems varied considerably depending on the carrier type (GOS vs. COS), the ratio used, and the presence of 15% silica (Table 7). The measurement of particle size allowed the characterization of the particle distribution in the resulting powders. The parameters Dx(10), Dx(50) and Dx(90) made it possible to assess the homogeneity of the systems and the influence of the type of carrier and silica addition on the powder structure. In GOS-based systems, increasing the amount of GOS led to a gradual decrease in particle size. Dx(90) values decreased from 241.2 µm in the ELD:GOS 1:1 sample to 56.088 µm in the 1:4 ratio, indicating finer particle formation. The addition of 15% silica further reduced particle sizes in all cases. For example, the Dx(90) in the 1:1 GOS system decreased to 17.945 µm after silica addition, showing improved powder homogeneity and fewer large agglomerates. COS-based systems without silica showed much higher Dx(50) and Dx(90) values, especially at lower COS ratios. The Dx(90) reached over 1300 µm in the 1:1 and 1:2 systems, indicating significant aggregation. However, the inclusion of silica led to a reduction in particle size. In the 1:1 COS system, Dx(90) decreased from 1319.98 µm to 48.69 µm, demonstrating the ability of silica to limit particle growth and improve uniformity even in systems prone to agglomeration. The results confirm that silica effectively reduces particle size and narrows the distribution, particularly in COS systems, contributing to more uniform and manageable powders. The stronger tendency of COS powders to agglomerate and to develop very broad particle-size distributions (Dx(90) > 1 mm at low carrier ratios) suggests less effective shell formation during droplet drying. Such heterogeneity increases local exposure to heat and oxygen and reduces encapsulation uniformity, which coheres with the lower post-drying anthocyanin contents measured for COS systems. The silica additive partly compensates for this by reducing cohesion and narrowing the size distribution, yet it does not equalize the protective capacity to that of GOS-based matrices.

Parameters such as inlet temperature, feed rate, and the concentration of carrier agents significantly influence the particle size of spray-dried powders. These process variables affect the rate of moisture evaporation, the extent of droplet shrinkage, and surface solidification dynamics. Elverrson et al. [58] found that higher extraction concentrations in spray drying led to an increase in particle size, thereby directly affecting the physical properties of the final product. Optimal microencapsulation of food bioactives typically results in particle sizes below 100 µm, which favor good dispersibility and stability [59]. In this context, our GOS-based powders with silica, showing Dx(90) between ~17–56 µm, fall well within the desirable range and demonstrate superior powder homogeneity. These findings confirm that the use of silica significantly reduces particle size, especially in COS systems, aligning our results with values considered optimal in spray-dried encapsulates. Kucharska-Guzik et al. [15] confirmed that the type of carrier, such as maltodextrin or inulin, has a significant impact on the particle size and morphology of spray-dried powders, highlighting the importance of carrier selection in microencapsulation processes.

#### 3.4.4. Density

Density is a critical parameter in spray-dried powders, as it influences not only flowability and compressibility but also storage stability, packing efficiency, and reconstitution behavior in final applications. Powders with higher true density typically show better handling properties and reduced susceptibility to moisture absorption and caking. In both GOS- and COS-based formulations, the presence of silica consistently led to denser powders compared to their respective control samples without silica (Table 8). This trend was observed across all extract-to-carrier ratios, indicating that the effect of silica on densification is robust and independent of formulation composition. The observed increase in density is likely due to improved particle packing, as silica particles fill the voids between larger extract and carrier components. This leads to a more compact powder structure with reduced internal porosity. The consistent observation that the addition of silica leads to higher density in both GOS- and COS-based formulations is noteworthy. Silica particles can fill void spaces between the larger particles of the extract and carrier, resulting in a more compact powder structure and reduced internal porosity. This densification mechanism aligns with findings from previous studies, which indicate that inorganic carriers like silica can enhance powder densification. In the case of ELD–GOS and ELD–COS powder systems, the inclusion of silica likely not only enhances density but also ensures that the powders exhibit optimal flow characteristics and stability during storage. The results across various extract-to-carrier ratios demonstrate that the densifying effect of silica is robust and independent of specific formulation compositions

### 3.5. Physicochemical Characterization of Systems

Due to their superior physicochemical characteristics, systems containing silica were selected for in-depth analysis. Differential scanning calorimetry (DSC), ATR-FTIR spectroscopy, and thermal degradation studies were performed exclusively on these formulations to assess their structural interactions and stability under thermal stress.

#### 3.5.1. ATR-FTIR Spectroscopy

**Figure 9:**
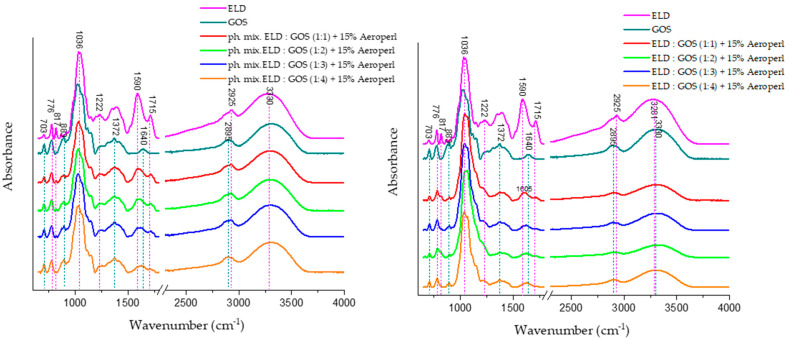
ATR-FTIR spectra of ELD:GOS spray-dried systems and corresponding physical mixtures with silica. Left panel: physical mixtures of elderberry extract (ELD) with galactooligosaccharides (GOS) at varying ratios (1:1 to 1:4) with silica. Right panel: spectra of spray-dried ELD:GOS formulations.

ATR-FTIR spectra presented in Figure 9 illustrate the characteristic absorbance bands of individual components and their mixtures. Physical mixtures and spray-dried systems containing ELD:GOS at various ratios with 15% silica are also shown.

The ELD spectrum reveals several distinct absorption bands associated with phenolic compounds. The band at ~1086 cm^−1^ is attributed to C–O stretching in phenolic groups, while bands at 1225 cm^−1^, 1372 cm^−1^, and 1450 cm^−1^ correspond to C–O–H bending, aromatic C–C stretching, and C–H deformation, respectively. Additionally, the broad band near 3300 cm^−1^ is indicative of O–H stretching, commonly associated with intermolecular hydrogen bonding in polyphenolic structures, confirming the presence of abundant hydroxyl-rich bioactive compounds in the elderberry extract.

GOS, in turn, exhibit characteristic bands in the 900–1200 cm^−1^ range due to C–O–C glycosidic bonds, and stretching vibrations of the pyranose ring are typically observed near 1020–1080 cm^−^**^1^**. These findings are consistent with previous literature, where ATR-FTIR analyses identified these regions as diagnostic for oligosaccharide- and polysaccharide-type carriers such as GOS.

In the spectra of spray-dried ELD:GOS systems (right panel), changes are most noticeable in the 3000–3600 cm^−1^ region. Specifically, the intensity of the broad O–H stretching band (~3300 cm^−1^) significantly decreases with increasing GOS content. This suggests a reduction in free hydrogen bonding interactions, likely due to the encapsulation of polyphenols within the GOS matrix and the possible formation of new intermolecular hydrogen bonds between extract and carrier.

Furthermore, the band near 1590 cm^−1^, attributed to C=C stretching in aromatic rings of polyphenols, shows both a shift and a decrease in intensity, indicating interactions or partial masking of polyphenolic structures during spray drying. Such spectral changes are indicative of molecular-level interactions between elderberry phenolics and the carrier matrix. Importantly, these spectral alterations are not observed in the physical mixture spectra (left panel), confirming that the modifications in band shape and intensity result specifically from the spray-drying process and the formation of structured delivery systems, rather than mere blending of components.

**Figure 10:**
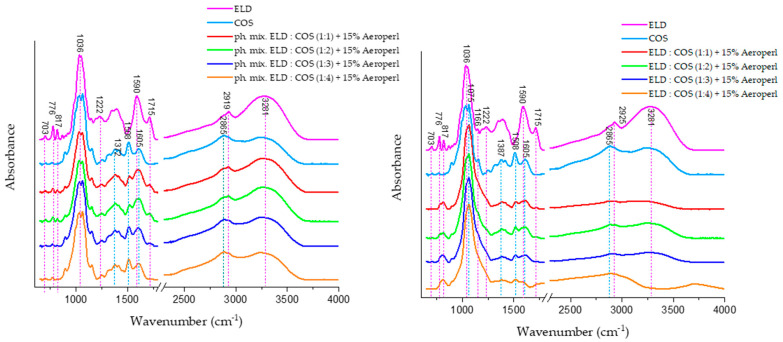
ATR-FTIR spectra of ELD:COS spray-dried systems and corresponding physical mixtures with silica. Left panel: physical mixtures of elderberry extract (ELD) with galactooligosaccharides (GOS) at varying ratios (1:1 to 1:4) with silica. Right panel: spectra of spray-dried ELD:COS formulations.

The ATR-FTIR spectra of chitosan oligosaccharide (COS) and its mixtures with elderberry extract (ELD) are shown in Figure 10. The COS spectrum displays characteristic bands consistent with previous reports in the literature. The absorption band near 1081 cm^−1^ corresponds to C–O–C stretching vibrations in the glucosamine ring, while bands at 875–895 cm^−1^ are attributed to C–H out-of-plane bending of β-glycosidic linkages. A broad band near 3300 cm^−1^ reflects O–H and N–H stretching vibrations, typical of the hydroxyl and amine groups present in COS. In the spray-dried ELD:COS systems (right panel), significant changes were observed in the hydrogen bonding region (3000–3600 cm^−1^). The broad O–H/N–H stretching band around 3300 cm^−1^ showed a marked decrease in intensity, indicating reduced hydrogen bonding due to encapsulation or interaction between elderberry polyphenols and COS. This is in contrast to the physical mixtures (left panel), where the O–H stretching band retains its intensity, suggesting a lack of molecular-level interactions.

Moreover, a distinct reduction in absorbance around 1387 cm^−1^ is visible in spray-dried systems, corresponding to CH bending vibrations and possible COO^−^ symmetrical stretching. This band’s attenuation is not observed in the physical mixtures, supporting the conclusion that structural reorganization and bonding occur during spray drying. Additionally, the region between 1508 and 1715 cm^−1^, attributed to aromatic C=C stretching (phenolic rings), C=O stretching of carboxylic and ketone groups, shows decreased intensity in spray-dried systems compared to physical mixtures. This implies possible encapsulation or interaction between polyphenols and COS, which may limit the accessibility or free vibration of these functional groups.

The observed spectral changes in the spray-dried systems, absent in the physical blends, further confirm that complexation or embedding of phenolic compounds within the COS matrix occurs during spray drying, contributing to the stabilization of the bioactive compounds.

#### 3.5.2. DSC

**Figure 11:**
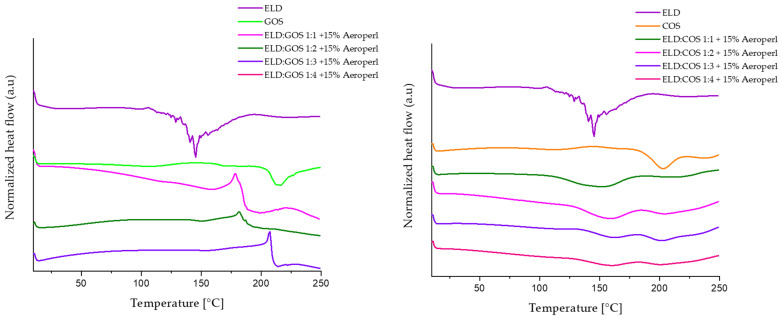
DSC thermograms of spray-dried powders containing elderberry (ELD) extract with galactooligosaccharides (GOS, left) and chitosan oligosaccharides (COS, right) at different ELD-to-carrier ratios (1:1 to 1:4) with 15% silica.

The effect of prebiotic carrier type and ratio on the thermal behavior of encapsulated elderberry (ELD) extract was evaluated by analyzing the DSC thermograms of pure ELD extract, GOS or COS carriers alone, and the spray-dried powders containing both components at different mass ratios (1:1 to 1:4) with 15% silica. Representative thermograms are shown in Figure 11. In the thermogram of pure ELD extract, several endothermic transitions were observed in the range of 50–150 °C, which are likely associated with the melting and/or thermal degradation of polyphenolic constituents, in particular anthocyanins and related glycosides. Similar patterns have been previously reported for thermolabile plant-derived phenolics [60,61].

The DSC thermograms demonstrate that the ratio of elderberry extract to prebiotic carrier significantly affects the thermal behavior of the spray-dried powders. In ELD:GOS systems, increasing the proportion of GOS from 1:1 to 1:4 results in a gradual shift in thermal transitions and a decrease in the intensity of endothermic peaks, suggesting improved thermal stability and stronger interactions between the extract and carrier. This enhanced stabilization by GOS is consistent with previous findings demonstrating their ability to form glassy matrices that limit molecular mobility and water-induced degradation, contributing to improved physical stability of encapsulated bioactives under thermal stress [62,63,64]. In contrast, thermograms of ELD:COS systems revealed a less consistent thermal behavior. The original ELD peaks were only partially suppressed, and new broad transitions emerged between 80 and 160 °C, without a clear shift with increasing COS content. This suggests weaker interaction between COS and the extract, possibly due to lower miscibility and fewer stabilizing interactions between the chitosan oligosaccharide matrix and polyphenolic compounds. This observation aligns with prior reports demonstrating limited thermal protection offered by chitosan-based carriers in encapsulation systems [65]. The absence of a clear, progressive shift in COS thermograms with increasing carrier content, together with the lower post-drying anthocyanin contents, less interactions on FT-IR analysis, and broader particle-size distributions, is consistent with less cohesive extract–matrix structuring in COS compared to GOS, which may contribute to weaker thermal stabilization of anthocyanins.

#### 3.5.3. Degradation Kinetics

Stability evaluation of spray-dried systems is essential for ensuring the long-term functionality of formulations containing thermolabile compounds such as anthocyanins and polyphenols. These bioactives are highly sensitive to elevated temperatures, moisture, and oxygen, which can lead to structural degradation and a reduction in antioxidant activity during storage or processing. To assess the resilience of encapsulated extracts, accelerated thermal conditions combined with kinetic modeling are widely used and offer predictive insight into their degradation behavior.

Several studies have demonstrated the effectiveness of degradation kinetics modeling as a tool to evaluate the protective capacity of encapsulating matrices. For example, Yu et al. [66] investigated the thermal degradation of anthocyanins from Rosa rugosa, encapsulated via both freeze drying and spray drying, and found that the process followed first-order degradation kinetics, with faster degradation occurring in spray-dried samples. The study highlighted the relevance of kinetic parameters, such as degradation rate constant (k) and half-life (t_0.5_), in comparing formulation performance under stress conditions.

Based on these findings, this study evaluated the degradation kinetics of spray-dried elderberry extract with GOS or CO at 85 °C for 4 h using a first-order kinetic model. Parameters *k*, *r*, and *t*_0.5_ were determined to quantitatively compare the protective effects of the carrier systems (Table 9 and Table 10). The degradation kinetics confirmed that GOS-based formulations significantly improved the thermal stability of elderberry extract, as evidenced by extended half-life values and reduced degradation rates with increasing GOS content. These results indicate that GOS is a more effective carrier for preserving polyphenols under thermal stress, likely due to its superior glass-forming and protective matrix properties. These findings are consistent with the DSC results, which demonstrated a shift in thermal transitions and reduced endothermic peak intensity in GOS-based systems, indicating stronger interactions between the extract and the carrier matrix. In addition, GOS molecules can form multiple hydrogen bonds with hydroxyl groups of anthocyanins, creating a protective microenvironment that minimizes pigment isomerization and structural rearrangements, as supported by previous encapsulation studies with oligosaccharides. In contrast, COS-based systems showed no substantial stabilizing effect, with half-life values comparable to or lower than the unprotected extract. Although COS contain functional groups capable of hydrogen bonding, their stabilizing effect on anthocyanins was limited. This may be due to their weaker ability to form amorphous, protective matrices compared to GOS. Furthermore, the polycationic nature of COS and the presence of amino groups may alter the local microenvironmental pH around anthocyanins, potentially accelerating degradation pathways [67]. DSC results confirmed the lack of strong molecular interactions in COS systems, as no significant shift in thermal transitions was observed. These findings suggest that COS are less effective under thermal stress, likely due to limited matrix formation and weaker polyphenol binding.

Recent studies have demonstrated that the choice of wall material, such as beta-glucan, inulin, pectin, or gum arabic, can significantly influence the stability of anthocyanins and vitamin C in spray-dried systems, with beta-glucan offering the highest retention of anthocyanins after storage [68]. Also cyclodextrins are widely used in the stabilization of anthocyanins and other phenolic compounds due to their ability to form inclusion complexes that protect bioactives from thermal, oxidative, and photodegradation [69,70,71,72,73]. Recent studies indicate that oligosaccharides can significantly impact the stability of anthocyanins. For example, research by Tobolka et al. [74] shows that anthocyanins glycosylated with oligosaccharides like rutinose exhibit greater protective effects against degradation compared to those with simple monosaccharides such as glucose. This difference can be attributed to the more complex structure of oligosaccharides, which can form more stable interactions with anthocyanins, thereby reducing their reactivity and susceptibility to degradation at elevated temperatures. The interaction of soluble small oligosaccharides derived from pectins also shows promise in stabilizing anthocyanins during processing. The study by Larsen et al. [75] demonstrates that oligosaccharides can complex with polyphenols like anthocyanins, potentially resulting in enhanced stability and bioavailability in food systems. The ability of these oligosaccharides to stabilize anthocyanins through hydrogen bonding and bridging effects is vital in developing effective preservation techniques. Lastly, the encapsulation of anthocyanins in oligosaccharide matrices, particularly with chitosan oligosaccharides, has been investigated for controlled release and enhanced stability. The findings reported by Lü et al. illustrate that systems with konjac glucomannan not only slow the release of anthocyanins but also maintain their stability against environmental challenges [76]. This encapsulation strategy shows potential for applications in functional foods and nutraceuticals aiming to prolong anthocyanin efficacy.

### 3.6. Innovation and Future Perspectives

Our findings provide new comparative insights into the use of prebiotic oligosaccharides as carriers in spray-dried plant extract systems. Although GOS have previously been incorporated into spray-drying matrices for other purposes, such as probiotic stabilization, there has been no prior side-by-side evaluation of GOS and COS for encapsulating anthocyanin-rich elderberry extract. The present results show that GOS-based systems achieved higher anthocyanin retention, better powder homogeneity, and greater thermal stability compared with COS-based systems. This comparative approach addresses a gap in the current literature and offers practical guidance for selecting oligosaccharide carriers in the design of multifunctional food and nutraceutical powders. To place our findings in the context of existing market solutions, it is worth noting several representative commercial elderberry-based formulations. ElderCraft^®^ spray-dried powders (Artemis International, Fort Wayne, IN, USA) are standardized to 4–15% anthocyanins and are designed for use in dietary supplements and functional beverages, yet they do not employ inherently prebiotic carriers. ElderMune™ (NutriScience Innovations, Milford, CT, USA) combines elderberry extract with the prebiotic fiber Sunfiber^®^ in a two-component system, whereas Probiotic+ Elderberry Boost (Akesi Wellness, Queensland, Australia) integrates elderberry anthocyanins with fructooligosaccharides and selected probiotic strains in a single blend. ImmunoMix Advanced Syrup (Aboca, Italy) delivers elderberry as part of a multi-botanical liquid formulation, and Natura Mix Advanced Energia (Aboca, Sansepolcro, Italy) presents orodispersible sachets containing anthocyanin-rich lyophilized fruit powders, including elderberry, alongside adaptogenic plant extracts. Compared with these products, the spray-dried systems developed in this work offer a distinct technological and functional advantage: the prebiotic component is embedded directly within the carrier matrix (GOS or COS), eliminating the need for separate prebiotic additives. Particularly, GOS-based systems demonstrated superior anthocyanin retention, improved powder homogeneity, and enhanced thermal stability relative not only to COS-based systems but also to the commercial formulations mentioned. This integrated approach improves the formulation process, enhances the synergistic effects of antioxidants and prebiotics, and may enable the creation of more stable, multifunctional nutraceutical products with competitive market potential.

Looking ahead, spray drying represents a robust and widely implemented technology in the food, nutraceutical, and pharmaceutical industries, which facilitates its translation into industrial practice. Critical considerations for large-scale production include feed formulation, energy efficiency, and minimizing powder losses in continuous operation. From a regulatory standpoint and economic consideration, the use of galactooligosaccharides (GOS) is particularly advantageous due to their GRAS status and established use in functional foods, whereas chitooligosaccharides (COS), despite their promising bioactivity, may require further toxicological and regulatory evaluation. GOS are already manufactured at industrial scale for infant formula and functional foods, which supports cost-effectiveness and availability, whereas COS remain less widely produced and therefore comparatively more costly [77].The inclusion of pharmaceutical-grade silica (Aeroperl^®^ 300 Pharma) is aligned with existing excipient frameworks, enhancing powder stability and flowability. Future work should therefore combine long-term stability studies with techno-economic and regulatory assessments to support the development of market-ready elderberry-based spray-dried systems.

## 4. Conclusions

Elderberry fruits are recognized for their high content of polyphenolic compounds, particularly anthocyanins, which contribute to their well-documented antioxidant and antidiabetic properties. The biological potential of elderberry varies depending on cultivar, maturation stage, and extraction method. Therefore, the development of effective delivery systems requires not only the appropriate selection of carriers but also precise optimization of encapsulation conditions to preserve the bioactivity of thermolabile constituents. In this study, galactooligosaccharides (GOS) proved to be superior to chitosan oligosaccharides (COS) as encapsulating matrices for spray-dried elderberry extracts. GOS-based systems exhibited enhanced thermal stability, as evidenced by extended degradation half-life values and favorable thermal transitions observed by DSC, indicating strong matrix–polyphenol interactions. In contrast, COS systems did not significantly improve stability, likely due to limited glass-forming ability and weaker molecular interactions. The inclusion of colloidal silica in all systems led to a consistent increase in powder density, attributed to improved particle packing, which contributed to enhanced powder appearance, including more uniform morphology and reduced agglomeration. These structural changes are advantageous for downstream handling, storage stability, and potential formulation into solid dosage forms. Altogether, the results support the application of GOS–silica matrices as an efficient platform for stabilizing polyphenol-rich elderberry extracts with antioxidant and antidiabetic potential. Future research should explore in vitro and in vivo models to confirm the bioefficacy of the encapsulated powders and evaluate their functional performance in food and nutraceutical systems. In summary, we deliver a comparative benchmark of prebiotic carriers, demonstrating that GOS provides superior anthocyanin protection and powder engineering outcomes versus COS for spray-dried elderberry extract, particularly when combined with silica. These findings clarify the role of carrier selection in designing multifunctional, antioxidant-prebiotic powders and provide actionable guidance for formulation of nutraceutical and pharmaceutical products where stability and manufacturability are critical.

## Figures and Tables

**Table 1:** Experimental conditions applied in the Box–Behnken design, including the input variables (methanol content, extraction time, and solvent-to-solid ratio).

Methanol Concentration (%)	Time of Extraction (min)	Ratio Liquid/Solid (mL/g)
0	15	60
100	15	60
0	90	60
100	90	60
0	52.5	20
100	52.5	20
0	52.5	100
100	52.5	100
50	15	20
50	90	20
50	15	100

**Table 2:** Composition of elderberry (ELD) extract systems with galactooligosaccharides (GOS) and chitooligosaccharides (COS) at various weight ratios, with or without the addition of 15% *w*/*w* Aeroperl 300 Pharma^®^ (Aeroperl).

Formulations with GOS (Galactooligosaccharides)	Formulations with COS (Chitoligosaccharides)
ELD:GOS (1:1)	ELD:COS (1:1)
ELD:GOS (1:2)	ELD:COS (1:2)
ELD:GOS (1:3)	ELD:COS (1:3)
ELD:GOS (1:4)	ELD:COS (1:4)
ELD:GOS (1:1) + 15% Aeroperl	ELD:COS (1:1) + 15% Aeroperl
ELD:GOS (1:2) + 15% Aeroperl	ELD:COS (1:2) + 15% Aeroperl
ELD:GOS (1:3) + 15% Aeroperl	ELD:COS (1:3) + 15% Aeroperl
ELD:GOS (1:4) + 15% Aeroperl	ELD:COS (1:4) + 15% Aeroperl

**Table 3:** Content of cyanidin-3-glucoside, cyanidin-3-sambubioside, and total polyphenols (TPC) in elderberry fruit extracts from different cultivars. Data are expressed as mean ± standard deviation (n = 3). Different letters within the same column indicate significant differences between means (*p* < 0.05; Tukey’s HSD test). n.d. = not detected.

Cultivar	Cyanidin-3-Glucoside	Cyanidin-3-Sambubioside	TPC
	(mg/g DW)	(mg/g DW)	(mg GAE/g DW)
Golden hybrid	n.d	n.d.	19.23 ± 2.38 ^a^
Samyl	0.215 ± 0.001 ^f^	0.335 ± 0.001 ^e^	43.34 ± 2.39 ^e^
Black beauty	0.017 ± 0.001 ^b^	0.073 ± 0.001 ^b^	36.58 ± 2.37 ^cd^
Bez dwubarwny	0.021 ± 0.002 ^b^	0.089 ± 0.002 ^c^	39.10 ± 1.77 ^d^
Samyl 1	0.163 ± 0.001 ^e^	0.846 ± 0.003 ^g^	57.75 ± 3.32 ^h^
Sampo	0.207 ± 0.002 ^g^	0.840 ± 0.004 ^g^	49.64 ± 1.15 ^f^
Obelisk	0.004 ± 0.002 ^a^	0.019 ± 0.003 ^a^	21.32 ± 3.06 ^a^
Bez koralowy	0.056 ± 0.006 ^c^	0.129 ± 0.006 ^d^	34.65 ± 2.16 ^c^
Sambo	0.161 ± 0.001 ^e^	0.436 ± 0.002 ^g^	48.13 ± 0.85 ^ef^
Haschberg	0.055 ± 0.001 ^c^	0.134 ± 0.002 ^d^	36.85 ± 2.20 ^cd^
Black tower	0.003 ± 0.001 ^a^	0.012 ± 0.001 ^a^	29.43 ± 1.80 ^b^
Haschberg 1	0.133 ± 0.006 ^d^	0.343 ± 0.001 ^e^	46.34 ± 1.65 ^ef^

**Table 5:** Moisture content (%) of spray-dried elderberry extract powders formulated with GOS or COS carriers at different ratios, with and without 15% silica addition. Data are expressed as mean ± standard deviation (n = 3). Different letters within the same column indicate statistically significant differences between means (*p* < 0.05; Tukey’s HSD test).

GOS Systems	Moisture Content (%)	COS Systems	Moisture Content (%)
ELD:GOS (1:1)	2.79 ± 0.13 ^a^	ELD:COS (1:1)	3.34 ± 0.22 ^a^
ELD:GOS (1:2)	7.12 ± 0.15 ^d^	ELD:COS (1:2)	5.72 ± 0.09 ^d^
ELD:GOS (1:3)	4.26 ± 0.11 ^b^	ELD:COS (1:3)	5.06 ± 0.09 ^c^
ELD:GOS (1:4)	5.76 ± 0.36 ^c^	ELD:COS (1:4)	5.93 ± 0.18 ^d^
ELD:GOS (1:1) + 15% Aeroperl	4.54 ± 0.07 ^b^	ELD:COS (1:1) + 15% Aeroperl	4.35 ± 0.23 ^b^
ELD:GOS (1:2) + 15% Aeroperl	2.54 ± 0.12 ^a^	ELD:COS (1:2) + 15% Aeroperl	3.99 ± 0.19 ^b^
ELD:GOS (1:3) + 15% Aeroperl	2.82 ± 0.28 ^a^	ELD:COS (1:3) + 15% Aeroperl	3.39 ± 0.25 ^a^
ELD:GOS (1:4) + 15% Aeroperl	5.23 ± 0.12 ^c^	ELD:COS (1:4) + 15% Aeroperl	4.94 ± 0.14 ^c^

**Table 6:** Content of cyanidin-3-sambubioside and cyanidin-3-glucoside in spray-dried powders. Values are expressed as mean ± standard deviation (µg/mg; n = 3). Theoretical values resulting from formulation composition are shown in parentheses.

System	Content of Cyanidin-3-Sambubioside (µg/mg)	Content of Cyanidin-3-Glucoside (µg/mg)
ELD	0.823 ± 0.040	0.247 ± 0.014
ELD:GOS 1:1	0.386 ± 0.011 (0.411 ± 0.020)	0.147 ± 0.020 (0.124 ± 0.007)
ELD:GOS 1:2	0.270 ± 0.008 (0.274 ± 0.013)	0.083 ± 0.016 (0.082 ± 0.005)
ELD:GOS 1:3	0.207 ± 0.014 (0.206 ± 0.010)	0.057 ± 0.008 (0.062 ± 0.004)
ELD:GOS 1:4	0.162 ± 0.006 (0.165 ± 0.008)	0.050 ± 0.014 (0.049 ± 0.003)
ELD:GOS 1:1 +15% Aeroperl	0.350 ± 0.018 (0.350 ± 0.017)	0.109 ± 0.035 (0.105 ± 0.006)
ELD:GOS 1:2 +15% Aeroperl	0.228 ± 0.004 (0.233 ± 0.011)	0.061 ± 0.006 (0.070 ± 0.004)
ELD:GOS 1:3 +15% Aeroperl	0.159 ± 0.009 (0.175 ± 0.009)	0.044 ± 0.005 (0.049 ± 0.003)
ELD:GOS 1:4 +15% Aeroperl	0.141 ± 0.001 (0.140 ± 0.007)	0.041 ± 0.009 (0.042 ± 0.002)
ELD: COS 1:1	0.316 ± 0.025 (0.411 ± 0.020)	0.082 ± 0.016 (0.124 ± 0.007)
ELD: COS 1:2	0.215 ± 0.004 (0.274 ± 0.013)	0.059 ± 0.003 (0.082 ± 0.005)
ELD: COS 1:3	0.147 ± 0.011 (0.206 ± 0.010)	0.040 ± 0.006 (0.062 ± 0.004)
ELD: COS 1:4	0.124 ± 0.010 (0.165 ± 0.008)	0.032 ± 0.007 (0.049 ± 0.003)
ELD: COS 1:1 +15% Aeroperl	0.318 ± 0.003 (0.350 ± 0.017)	0.088 ± 0.012 (0.105 ± 0.006)
ELD: COS 1:2 +15% Aeroperl	0.188 ± 0.004 (0.233 ± 0.011)	0.049 ± 0.001 (0.070 ± 0.004)
ELD: COS 1:3 +15% Aeroperl	0.140 ± 0.002 (0.175 ± 0.009)	0.038 ± 0.002 (0.049 ± 0.003)
ELD: COS 1:4 +15% Aeroperl	0.118 ± 0.001 (0.140 ± 0.007)	0.031 ± 0.002 (0.042 ± 0.002)

**Table 7:** Particle size distribution (Dx10, Dx50, Dx90) of elderberry extract–carrier system. Data are expressed as mean ± standard deviation (n = 5). Different letters within the same column indicate statistically significant differences between means (*p* < 0.05; Tukey’s HSD test or Games–Howell test in the case of unequal variances), calculated separately for GOS and COS systems.

System	Particle Size (µm)		
	Dx (10)	Dx (50)	Dx (90)
ELD GOS 1:1	3.0 ± 0.1 ^c^	14.5 ± 0.2 ^e^	241.2 ± 2.7 ^f^
ELD GOS 1:2	1.6 ± 0.0 ^a^	5.6 ± 0.2 ^b^	134.5 ± 10.6 ^b^
ELD GOS 1:3	1.6 ± 0.1 ^a^	5.1 ± 0.5 ^a,b^	96.3 ± 16.7 ^a^
ELD GOS 1:4	1.6 ± 0.0 ^a^	10.9 ± 0.1 ^a^	35.0 ± 1.3 ^a^
ELD GOS 1:1 +15% Aeroperl	2.8 ± 0.0 ^c^	13.1 ± 0.3 ^d^	56.1 ± 1.2 ^c^
ELD GOS 1:2 +15% Aeroperl	2.5 ± 0.0 ^b^	11.3 ± 0.3 ^c^	49.1 ± 1.8 ^a^
ELD GOS 1:3 +15% Aeroperl	2.3 ± 0.0 ^b^	13.9 ± 0.0 ^e^	55.7 ± 1.0 ^a^
ELD GOS 1:4 +15% Aeroperl	3.5 ± 0.1 ^d^	4.9 ± 0.0 ^c^	18.0 ± 2.9 ^a^
ELD COS 1:1	4.0 ± 0.8 ^b^	156.4 ± 41.3 ^d^	1320.0 ± 81.3 ^f^
ELD COS 1:2	4.0 ± 0.1 ^b^	144.3 ± 8.3 ^d^	1313.1 ± 88.4 ^e^
ELD COS 1:3	2.5 ± 0.2 ^a^	10.4 ± 3.7 ^abc^	1145.2 ± 127.6 ^d^
ELD COS 1:4	2.3 ± 0.0 ^a^	6.1 ± 0.1 ^a^	14.8 ± 0.5 ^a^
ELD COS 1:1 +15% Aeroperl	4.4 ± 0.1 ^b^	13.6 ± 0.2 ^c^	48.7 ± 1.8 ^c^
ELD COS 1:2 +15% Aeroperl	4.2 ± 0.1 ^b^	13.3 ± 0.1 ^b^	46.8 ± 1.3^,c^
ELD COS 1:3 +15% Aeroperl	4.4 ± 0.1 ^b^	14.2 ± 0.2 ^c^	52.1 ± 1.2 ^c^
ELD COS 1:4 +15% Aeroperl	3.8 ± 0.1 ^b^	12.5 ± 0.1 ^b^	50.2 ± 0.6 ^b^

**Table 8:** True density (g/cm^3^) of ELD–GOS and ELD–COS powder systems. Data are expressed as mean ± standard deviation (n = 3). Different letters within the same column indicate statistically significant differences between means (*p* < 0.05; Tukey’s HSD test).

GOS Systems	Density (g/cm^3^)	COS Systems	Density (g/cm^3^)
ELD:GOS (1:1)	1.5418 ± 0.0012 ^b^	ELD:COS (1:1)	1.5253 ± 0.0015 ^c^
ELD:GOS (1:2)	1.5491 ± 0.0023 ^c^	ELD:COS (1:2)	1.5175 ± 0.0015 ^bc^
ELD:GOS (1:3)	1.5267 ± 0.0019 ^a^	ELD:COS (1:3)	1.5102 ± 0.0012 ^b^
ELD:GOS (1:4)	1.5262 ± 0.0010 ^a^	ELD:COS (1:4)	1.4697 ± 0.007 ^a^
ELD:GOS (1:1) + 15% Aeroperl	1.5968 ± 0.0029 ^e^	ELD:COS (1:1) + 15% Aeroperl	1.5800 ± 0.0021 ^f^
ELD:GOS (1:2) + 15% Aeroperl	1.6083 ± 0.0041 ^f^	ELD:COS (1:2) + 15% Aeroperl	1.5583 ± 0.0056 ^ef^
ELD:GOS (1:3) + 15% Aeroperl	1.5739 ± 0.0010 ^de^	ELD:COS (1:3) + 15% Aeroperl	1.5701 ± 0.0020 ^d^
ELD:GOS (1:4) + 15% Aeroperl	1.5677 ± 0.0024 ^d^	ELD:COS (1:4) + 15% Aeroperl	1.5347 ± 0.0024 ^e^

**Table 9:** Degradation kinetics of ELD–GOS systems with 15% silica at 85 °C. k—degradation rate constant; r^2^—coefficient of determination; t_0.5_—half-life (h). Values of t_0.5_ are expressed as mean ± standard deviation (n = 3).

System	k (min^−1^)	r^2^	t_0.5_ ± SD (h)
ELD	18.328	0.965	0.631 ± 0.035
ELD:GOS (1:1) + 15% Aeroperl	9.27	0.95	1.245 ± 0.001
ELD:GOS 1:2 + 15% Aeroperl	5.481	0.978	1.891 ± 0.093
ELD:GOS 1:3 + 15% Aeroperl	4.293	0.979	2.366 ± 0.137
ELD:GOS 1:4 + 15% Aeroperl	3.226	0.964	3.082 ± 0.205

**Table 10:** Degradation kinetics (k, r^2^, t_0.5_) of ELD–COS systems with 15% silica at 85 °C. k—degradation rate constant; r^2^—coefficient of determination; t_0.5_—half-life (h). Values of t_0.5_ are expressed as mean ± standard deviation (n = 3).

System	k (min^−1^)	r^2^	t_0.5_ ± SD (h)
ELD	18.328	0.965	0.631 ± 0.035
ELD:COS 1:1 + 15% Aeroperl	18.925	0.946	0.616 ± 0.003
ELD:COS 1:2 + 15% Aeroperl	18.923	0.892	0.613 ± 0.001
ELD:COS 1:3 + 15% Aeroperl	20.318	0.891	0.559 ± 0.005
ELD:COS 1:4 + 15% Aeroperl	16.414	0.927	0.700 ± 0.002

## Data Availability

The data supporting the findings of this study will be made available on the Zenodo repository under the title of the publication.

## Abbreviations

The following abbreviations are used in this manuscript:

AGEs	Advanced Glycation End Products
ATR-FTIR	Attenuated Total Reflectance Fourier-Transform Infrared Spectroscopy
COS	Chitooligosaccharides
CUPRAC	Cupric Ion Reducing Antioxidant Capacity
DSC	Differential Scanning Calorimetry
DPPH	2,2-Diphenyl-1-picrylhydrazyl
ELD	Elderberry Extract
FOS	Fructooligosaccharides
FRAP	Ferric Reducing Antioxidant Power
GOS	Galactooligosaccharides
HPLC-DAD	High-Performance Liquid Chromatography with Diode-Array Detection
IC_0.5_	Inhibitory Concentration at 0.5 Absorbance
PBS	Phosphate-Buffered Saline
SCFAs	Short-Chain Fatty Acids
TGA	Thermogravimetric Analysis
TPC	Total Polyphenol Content
T2DM	Type 2 Diabetes Mellitus
